# Exploring the Needs and Concerns of Women with Early Breast Cancer during Chemotherapy: Valued Outcomes during a Course of Traditional Acupuncture

**DOI:** 10.1155/2013/165891

**Published:** 2013-09-02

**Authors:** S. Price, A. F. Long, M. Godfrey

**Affiliations:** School of Healthcare, University of Leeds, Baines Wing, Leeds LS2 9JT, UK

## Abstract

Women diagnosed with breast cancer experience symptom clusters in addition to existential issues from a life-threatening diagnosis during chemotherapy. A complementary therapy, such as traditional acupuncture (TA) with its whole-person orientation, may help to modify these effects, alongside inducing other patient benefits. Exploring the needs and concerns of women and perceived benefits of TA would add to knowledge about its integrative treatment potential. *Methods*. A longitudinal qualitative study recruited fourteen women to receive up to ten sessions of TA during chemotherapy. They were interviewed before, during, and after chemotherapy. Two practitioners of TA delivered treatment and were interviewed before and after the study, and kept treatment logs and diaries. Interviews were recorded and transcribed, and the data were analysed using grounded theory. *Findings*. Both broad and specific benefits were reported by the women; a highly valued outcome was enabling coping through the alleviation of symptoms and increased well-being. Practitioners dealt with the presented symptom clusters facilitating outcome patterns, including and beyond individual symptom changes. Further research on TA as a flexible intervention able to respond to the changing needs and concerns of woman during chemotherapy along with the measure of such outcome patterns is warranted.

## 1. Introduction 

Breast cancer is the most common cancer in women, accounting for nearly one-third of all new cancers for women. In the UK in 2010, there were 11,556 deaths from breast cancer, and a woman has a one in eight chance of a breast cancer diagnosis in her lifetime [1]. Although breast cancer is characteristic of a chronic condition, it may be experienced at different points as chronic interspersed with acute episodes, requiring multiple, intensive interventions [2]. Women diagnosed with breast cancer are likely to very quickly undergo surgery, then chemotherapy followed possibly by other adjuvant treatments such as Herceptin, radiotherapy, or Tamoxifen which extends treatment over months and increasingly years. The speed of the diagnostic and treatment trajectory in breast cancer reflects evidence on the significance of early diagnosis and treatment on survival. In the UK pursuit of a two-week referral pathway from presentation of suspect symptoms to specialist diagnosis and a maximum 31-day wait from diagnosis to first treatment for all cancers [3]. 

The speed along with the disorientating shock of diagnosis and initial treatment may induce additional problems alongside the common “side-effects” already known to arise from surgery and chemotherapy. There is thus considerable potential for a complementary therapy, such as traditional acupuncture (TA) with its whole-person approach [4], to help modify these effects and to potentially induce other patient benefits, for example, improvement in well-being [4, 5] and cancer-related fatigue [6]. Given the complex combination of factors, it is pertinent to uncover the needs and concerns of women and perceived benefits of TA, so as to be able to offer additional acceptable and meaningful support at this time.

This study focuses on a group of women with early breast cancer (EBC). EBC is defined as cancer which is only in the breast and the lymph nodes nearby, has not spread to other parts of the body or grown directly into nearby tissue [1]. The research literature documents a wide range of symptoms and treatment side-effects for EBC and their occurrence in clusters or combinations that may vary over the illness course. Cancer-related fatigue (CRF) is the most prevalent and problematic symptom; it is well described as a “persistent, subjective sense of tiredness related to cancer and cancer treatment that interferes with usual functioning” [7]. Although CRF is multidimensional, its physiological mechanisms are unknown [8], making treatment for it elusive. CRF does not however occur in isolation but rather as one component of a cluster of symptoms that together exacerbate distress [9, 10]. Other symptoms, including emotional distress, hot flushes, night sweats, insomnia, and pain, are frequently clustered together, with many linked to CRF [9, 11–18].

While many researchers have explored the prevalence of symptom clusters, there is little research to date which directly examines patient needs at this time or the benefit of possible supportive interventions. Symptom clusters are an even greater problem as multiple strands of side-effects and symptoms create multi-dimensional effects for the individual. More understanding of symptom clusters may lead to the identification and use of outcome measures that capture the totality of the experience. 

This paper reports on findings from a prospective qualitative study for women diagnosed with EBC, receiving a course of TA treatment alongside receipt of chemotherapy. The focus of the paper lies on exploring women's perceived benefits arising from TA and practitioner desired outcomes. In so doing, the paper demonstrates the significance of TA practitioners' treating patient concerns, the notions of symptom clusters and outcome patterns and a resultant need for studies to measure such outcome patterns. 

## 2. Materials and Methods

A qualitative longitudinal study was developed to offer women who had recently been diagnosed with EBC the opportunity to access, via their oncologist, up to ten sessions of TA while they underwent chemotherapy [19]. Fourteen women with EBC were recruited from two NHS hospital trusts in the UK, the sample size being restricted due to funding constraints and intensive in-depth interviews undertaken over time. Two experienced acupuncturists provided the TA sessions, at one of four centres, including one private practice, and were asked to approach treatment as they normally would, thus replicating real-world practice (e.g., individualised treatment responding to the individual at each treatment). The acupuncturists were both members of the British Acupuncture Council, the accrediting body for TA in the UK. 

In-depth interviews were conducted with the women before, during and after (within six months of finishing) chemotherapy treatment while they received TA. The TA practitioners were interviewed before and after completion of all TA treatment for participants and asked to keep treatment logs and diaries. Treatment logs provided in-depth understanding of practitioner reflections on what they did and why, a contemporaneous picture of their actual practice with individual women and any new problems experienced by their patients which resulted in altered treatment priorities. Their diaries offered reflections on treatment-related issues. 

In-depth interviews, lasting between 50 and 120 minutes, were conducted, recorded, and transcribed. All participants were given a pseudonym. Data were analysed using a grounded theory approach (led by SP); this included simultaneous data collection and analysis, open coding, and memo-writing, leading onto focussed coding and the development of key categories [20] using the method of constant comparison [21]. Field notes were made immediately after each interview including methodological, observational and reflexive notes. A reflexive stance was maintained throughout the study. The diaries and treatment logs were included in this process. The on-going analysis was checked for inclusiveness and consistency with coauthors, and the data searched for disconfirming cases. As befits a grounded theory approach [22] focus lay on process and change. For example, attention was directed at understanding how practitioners assessed and responded to women's articulation of symptoms and experience of illness as these changed over time and in relation to the treatments and how women in turn experienced acupuncture in the context of the meaning and experience of breast cancer at this stage in the illness trajectory. Ethical approval was obtained from the local REC (REC approval number reference: 07/H1306/79). Only the findings relating to practitioner and patient valued outcomes and perceived benefits are reported in this paper. 

## 3. Results and Discussion

The mean age of the women was 54 (range of 41–76). Six had dependents, either elderly parents or children under the age of 16 living at home. All the participants but one had a household income of less than *£*30,000, and two had degree or equivalent educational status. All the participants had had surgery (two had a mastectomy), and all received six cycles of EPI-CMF chemotherapy, begun just before their receipt of TA. The findings reveal the range of concerns presented by the women to the practitioners largely dominated by an overarching experience of emotional and physical distress. These experiences included chronic long-standing problems and acute infections, as well as a range of perceived benefits. 

### 3.1. Practitioner Recordings of Patient Reported Concerns

The main areas of patient concern were those of fatigue and emotional upset along with both sleep disturbance from heat and night sweats and distress or anxiety. These combinations appeared to come and go in order of importance for individuals and at different stages of the treatment trajectory.  Table 1 represents a summary of the main complaints or concerns recorded by practitioners in their treatment logs. 

The logs, while only having space to record the first two problems of each treatment, and practitioner and patient interviews all demonstrated that the women reported several problems occurring simultaneously and shifting in importance from day to day and from treatment session to treatment session. At times, women appeared to have more extreme fatigue manifesting as exhaustion and “*disorientation*” with weakness and consequent emotional upset. Most of the time most of the women experienced a minimum of two important concerns; in Table 1 the most common symptom clusters (simultaneously occurring) were fatigue, emotional distress, night sweats, insomnia, and digestive problems including nausea.

Occasionally (mentioned 17 times in a total of 128 treatments) they had a “*good*” week and had nothing major to complain about. In the diaries, practitioners recorded dilemmas they faced over how to approach treatment because of the range of problems presented, both chronic and acute, and TA's focus on treating the whole person in the context of their life. Examples include two participants going through very painful divorces and others having elderly parents and children depending on them. Their life-world encompassed the perceived life threat of the cancer, difficulties many experienced in talking through their fears with those close to them, and the strongly held view that positivity in the face of the cancer was critical to recovery, even for those with a supportive network. Talking about and sharing these issues with the practitioners occurred gradually over time in the multiple treatments and within the differential diagnosis process. This enabled the practitioner to draw out what would be most beneficial to work on in the immediate term that would affect change in relation to whole person issues. In these difficult circumstances, all three sources of data confirmed that the practitioner focussed on the whole person and the constellation of symptoms, not just a single symptom, onto the achievement of a set of outcomes/outcome patterns. 

### 3.2. Practitioners' Desired Outcomes

The practitioners pragmatically addressed the multiple problems presented to them that were both acute and chronic. They wanted to achieve broad effects relating to the whole person and longer term outcomes such as “*enable coping*,” as well as the immediate relief of symptoms. A major aspect of their focus was on “*resolving outcome patterns*” in response to the presentation of women's symptom clusters. Outcomes were seen to be linked together to loosely reflect the *patterns* of imbalance, according to the theory of Traditional Chinese Medicine (TCM), perceived during the differential diagnosis [23]. In asking one of the practitioners, Helen, what kinds of things acupuncture helped with, she responded:

*Everything. I think the emotional level and the physical level. I do think we have pre-empted chemo symptoms—I think we have controlled past patterns and therefore pre-empted worsening chemo states. *

**Figure 1:**
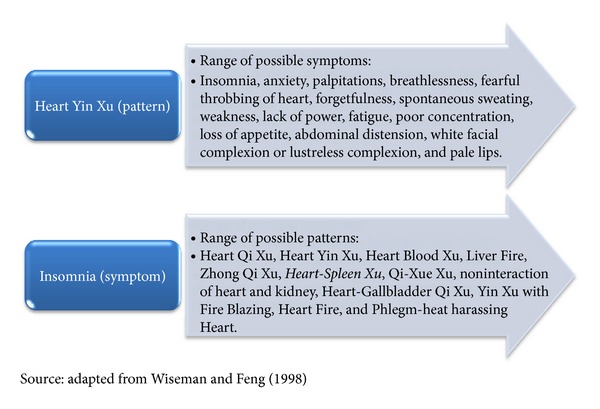
Pattern and symptom: relationships according to TCM for insomnia.

She added that acupuncture was preventative and that by addressing *patterns* a range of symptoms could be minimised or avoided. The word “*patterns*” (*bian zheng*) has specific meaning in TCM theory: as a set of specific collections of signs and symptoms. An example is presented in Figure 1, demonstrating how the whole system approach in the theory of TCM (*pattern differentiation*) and guiding the practitioners' treatments links across to patient experiences of multiple symptoms, in this case anxiety, together with insomnia, fatigue, and other symptoms.

Helen went on to articulate the complex process of the experience of a cancer diagnosis and how both the acupuncture and the care helped the person.

*I think it is difficult to be specific but I think any illness brings up the previous emotional patterns, it's not just the emotions about the cancer—it's about you know “is my husband being supportive” or for some “I have been looking after my father for all that time and rushing here there and everywhere” and how much has that contributed. So I think it helps the person—the cancer has catalysed their mind so that they can express that, the sessions help them express it more and the acupuncture helps them deal with it I think. *

This extended extract points to different influences at work within a person: firstly, the way a person is (in the past and now) in their *patterns*; secondly, the profound effect of the cancer diagnosis; thirdly, the acupuncture sessions providing an opportunity for an expression of all of these things; and, finally, the acupuncture treatment having an effect that helps the person deal with it all. As Helen outlined, in treating the (whole) person, it was not just about the cancer but about life before the diagnosis and how the impact of the diagnosis might focus or reawaken past issues. By addressing *patterns* in TCM theory and addressing different dimensions of health all at once, both practitioners spoke consistently of affecting change in the whole person, physically, emotionally, and psychologically. This is made explicit in Figure 1 showing the difference between *pattern differentiation* and symptoms. 

The other practitioner, Diane, reported the experience of a participant, Kathy, who was astonished that the uncharacteristic anxiety, experienced since her diagnosis, had resolved after the first TA treatment. Just as Diane used the example of Kathy's anxiety resolving quickly, Kathy in her interview spontaneously reported her astonishment as she was ironically concerned that the acupuncture could have had such an effect, because she was so altered.

*And what had happened? I explained how the acupuncture had worked because it had obviously just smoothed things out grounded {Kathy} and she was sleeping and she was really quite chilled out about the whole cancer problem in a way to how she had been. She had not been sleeping, she had been having sweats—sweats because of her anxiety nothing else, so she was really disconcerted as to what on earth had happened and I was just— great…fantastic! *

In this example, it is not just Kathy's anxiety that had improved so quickly but her sleep and her night sweats. As illustrated in Figure 1, through Diane treating the pattern of *Heart-Spleen Xu*, she was able to achieve this range of outcomes. Diane believed this was an effect of the acupuncture treatment itself addressing clusters of symptoms simultaneously.

### 3.3. Patient Perceived and Reported Benefits of TA

Nearly all the women felt they had coped well and certainly better than they expected to, despite all of their problems. They perceived both broad and specific benefits; some attributed this directly to the acupuncture because changes were felt immediately after each treatment. For example, Liz described a trip to the local café having had acupuncture earlier in the day, beforehand feeling a combination of fatigue, nausea, and indigestion and realised with exclamation as she tucked into a scone how she felt completely transformed. She went on to say

*I found it very peaceful, very relaxing, very calming, and as I say it was just a great help to know that on a weekly basis I could go to that lovely quiet room, and talk to somebody and have treatment that I feel was helping with the side-effects of the chemo.*

Moreover, in looking back months after the chemotherapy had finished, they had a sense of a longer term effect; time gave the women a perspective on the whole treatment experience. Others found it difficult to attribute benefit directly to the TA. In part, this was because the chemotherapy itself was a new, but invasive, experience. TA also involved a new experience. One participant expressed explicitly that she did not know how acupuncture could work nor have such effects. 

While practitioners had spoken substantially about using acupuncture to increase vitality and fortifying and strengthening the person to enable them to cope better, the women reported that this “*feeling better*” could be about symptom relief (reported by most of the participants), combined with inducing a sense of calm or feeling more relaxed. They talked about feeling more composed, balanced, lighter, energised and better in themselves as well as more peaceful. For example, one of the women, Anne, commented:

*I don't know what you are supposed to feel from acupuncture, because it is the first time I had ever had that, but I know that when I used to leave from the session, I used to feel good in myself. Like, feel good about myself, and feel like I'd got energy, and I didn't feel tired.*

This general benefit may be an important outcome for women who are receiving medical interventions that are in some way bringing about reduced well-being and vitality. All the participants reported that they looked forward to each session; regardless of what they felt the acupuncture was doing for them, it was an enjoyable experience. 

To aid analysis and interpretation, the set of specific symptoms women reported during each interview being eased by the TA are summarised (see Table 2). During the interviews, all the women, without exception, discussed these specific symptoms being helped by the acupuncture. Some participants described feeling very unwell at times, often with a combination of extreme tiredness, nausea, and a disorientated feeling that came on unpredictably. Others rattled off whole lists of symptoms they experienced and found acupuncture helped with. It was the immediate relief of these symptoms that convinced women that acupuncture worked. Some women suffered from extreme night sweats and reported their frequency and intensity reducing immediately. Night sweats went hand in hand with disturbed sleep which in turn contributed to fatigue. Several women reported their sleep was improved and that they felt more relaxed at night. Symptoms that seemed unrelated were also reported as having being eased and resolved. Jane gave an example as she experienced combinations of fatigue, joint pain, headaches, digestive discomfort, and bloating. 

Several participants talked about help with headaches and eye pain and others about help with emotional problems, or their fear of not coping emotionally. Reduction in anxiety levels was dramatic for two participants, Lindsay and Kathy, almost immediately on starting acupuncture; for another, Anne, her fear of depression returning was managed as illustrated below by the acupuncture.

*It's kept me bubbly. Yeah it's kept me bubbly because otherwise I would have been down…Because I have suffered with depression quite badly. So I don't want to go down that road. Because it's like a no return sometimes, you can get that far down, so I prefer to stay at the high notes, and be all bubbly-bubbly…So I never got down, I never got depressed.*

There was a high attendance for the acupuncture treatments, with all participants looking forward to and enjoying the whole experience of the acupuncture treatment, the interaction with the practitioner, and the immediate benefit that most of them believed they would get. For several participants, the contexts of their lives were problematic, embracing the roller coaster pattern of the illness trajectory: that is, becoming a cancer patient overnight, having challenging treatment, the diagnosis itself, and complex relationships with others in their lives. In the acupuncture session, they could both talk about ordinary things where the cancer did not define them and express their fears and vulnerabilities. Acupuncture sessions provided light relief and a safe haven, as well as physical or psychological benefit. In addition they could get “*topped up*” with something enabling them, as several participants reported, to have enough energy after the session to go into town and shop or be alone. 

Enabling coping emerged as both a broad and possibly longer term valued effect of the acupuncture care. Several participants reported this effect at both the second and third interviews. The acupuncture seemed to be able to treat different symptoms in one session and also produce whole person effects, such as feeling stronger, feeling healthier and feeling more able to cope. Coping could result from an alleviation of symptoms as well as improved well-being. For instance, Vera, who had comorbidity and chronic pain, was able to stop taking painkillers and reduce other medication during the period of time that she had acupuncture. She felt a new lease of life and reported that she had never felt better, even though at this time she was having chemotherapy. Acupuncture seemed to lead to an improved mental outlook which made other symptoms feel more bearable.

Being “able to buy” space and time to process the meaning of the diagnosis enabled the women to develop resources to put on a brave face for others and carry on as normal or, in other words, find a way of coping. For example, one of the women, Lena, reported facing many difficulties in accepting and adjusting to the diagnosis and subsequent surgery and experienced debilitating fatigue at times. She felt the whole experience of acupuncture helped her to cope; as illustrated below, a key part of what made her believe in its leading to benefits was that she felt a lot worse when she stopped having TA.

*I think comparing when I was having it plus the chemo and then after stopping it, well, whilst I was having it, I think it helped me looking back, I think it has helped me a lot, to keep all the symptoms at bay—you know all that I complain of. Although it was there and I felt sick, and I felt tired, and I was able to cope. (But) because after the sessions of acupuncture finished, I suffered badly and I put it down to not having the acupuncture to help me. *

Looking across these findings from this small-scale, qualitative longitudinal study of the provision of TA for women during chemotherapy a wide range of patient concerns have been revealed. The TA practitioners focused treatment on both specific and sets of symptoms, with a view to generate broader effects such as enabling coping. The women all valued the TA, experiencing both specific and more general benefits which in many instances they attributed directly to the TA treatment. Throughout, it was apparent that the concept of symptom clusters, prevalent in the literature, was supported by the triangulated study data, appearing in both sets of interviews, in the treatment logs and as concerns in the diaries. Combinations of symptoms added up to, at times, a sense of total unwellness and collapse. TA appeared to have great flexibility in addressing symptom clusters that had both acute and chronic features. 

The notion of “outcome patterns” emerged from the data where collections of signs and symptoms in a *pattern of imbalance*, identified in the TCM differential diagnosis within each treatment session and the wider context of a developing, strong therapeutic relationship between the woman and practitioner. Treatment affected changes in these patterns and resulted in a set of interlinked patient-experienced and perceived benefits. These outcome patterns reflected TA's whole person approach in treatment, with changes experienced in the whole person. Such outcome patterns are collectively interrelated according to this TCM theory and not discrete. This is an important point as the majority of research on acupuncture involves use of a standardised set of acupuncture points for a fixed symptom and thus, if generalised to TA, whose practice is not reflected in use of a set prescription, may reduce and/or underestimate the effects of TA and its potential to focus on multiple and interrelated problems. 

The study's triangulated data demonstrates that the valued multiple effects of TA come about by design and are inherent within its theory of change [19, 23], rather than just fortuitous side-effects. This suggests the problematic nature of the common use of single outcome measures as a primary outcome in many TA research trials; no single outcome measure has the breadth or flexibility to capture this range of perceived benefits. Finding, and or developing outcome measures that capture all the interrelated changes, which are also tied up with the process of care, pose an important task for TA and other CAM researchers [24–28]. 

Practitioners wanted to *strengthen and fortify* their patients as they went through chemotherapy. Other studies have reported perceived benefits by patients, including increases in personal energy and in emotional and physical strength [29]. Other literature on CAM also points to perceived/reported changes to energy and strength [28, 30]. A broad intended outcome of the practitioners in the EBC study was to enable coping. This was brought about by using acupuncture to reduce the impact of symptoms both directly and through *strengthening and fortifying*. These two intended outcomes fed into one another; as the women reported feeling more able to cope and their sense of well-being and confidence increased. 

Further research is needed to add to and confirm the findings of this small-scale study. This could valuably focus on exploring how TA enables coping in the face of uncertainty for those with breast cancer or other conditions with both acute and chronic features and whether, and how enhanced coping is related to vitality. Of perhaps greatest importance is the need to develop outcome tools that measure change over time for a whole-person treatment approach and resultant whole-person effects, in particular, to measure outcome patterns. A similar finding was reported in our review of acupuncture research for symptoms common to breast cancer sufferers [23]. Seers et al. (2009) [30] found that the concerns of people using CAM while suffering from cancer ranged over thirty categories. Their study used the Measure Yourself Concerns and Well-being (MYCaW) instrument [31] to record change, and a significant improvement was shown overall. Finding ways of capturing change where problems range from symptom clusters to changes in self-concept is not easy, but the MYCaW instrument may be a useful way of capturing a range of changes important to the person and provides also opportunity to collect some narrative. One proviso might be that, when patients have a recent diagnosis and rapidly undergo treatment, needs and concerns will fluctuate wildly and may also be difficult to identify.

Care must be taken in generalising from this study, given its sample size due to funding constraints, resulting in a limited number of women participants able to receive the TA sessions and their provision by one of two TA practitioners. However, the internal validity of this study is strengthened through its multiple points of interview (for both the women and practitioners) and use of practitioner treatment logs and diaries. Most importantly the study was a pragmatic one, conducted to explore and reproduce real-world practice. This was evident in the role of the oncologists who prioritised certain patients over others to refer for TA and thus into this study and the TA practitioners being asked to diagnose and treat the referred women according to the principles of TCM and TA.

## 4. Conclusions

Women experience a range of debilitating symptoms during chemotherapy that fluctuate unpredictably and range from existential issues to multiple symptom clusters. TA was found to be a flexible intervention with a focus on the person in their life-world context as well as providing immediate and on-going relief of acute symptoms. The findings demonstrated TA practitioners' treating patient concerns, leading onto the achievement of broader, nonsymptom specific outcomes. The women valued the whole experience of TA and reported receiving considerable benefit from it, providing further evidence of TA as a supportive treatment in an integrative manner for women during chemotherapy. 

## Figures and Tables

**Table 1:** Summary of main and secondary concerns recorded in treatment logs*.

Main complaint	Secondary complaints
Tiredness/fatigue/exhaustion (27)	Night sweats/hot flushes (29)
Emotional (stress/distress/anxiety) (18)	Fatigue/tiredness/weakness (22)
Night sweats/hot flushes (12)	Emotional (stress/distress/anxiety) (17)
Nausea (11)	Poor sleep (19)
Pain—gastric, arthritic, tension (9)	Heartburn/gastritis (10)
Depression and feeling low (7)	Aches and pains including pain in chemotherapy arm, pain due to tension, and chest pain (10)
Ear problems (pain and deafness, tinnitus, and infection) (6)	Eyes—blepharitis (dry/pain/infection/red/inflamed) (7)
Anxiety/fear (5)	Sore mouth/throat (7)
Poor sleep (4)	Nausea/poor appetite (7)
Migraine/headache (4)	Breathless/tight chest (5)
Sore throat/mouth (3)	Disorientation/“spaced out”/feeling disconnected (4)
Head cold/cough (2)	Headache (4)
Constipation (2)	Breast pain or tenderness or infection or fluid (5)
Disorientation/“spaced out”/feeling disconnected (1)	Diarrhoea (5) constipation (3)
Fluid around wound (1)	Oedema (5)
	Back pain (4)
	Blood glucose erratic (4)
	Tinnitus (3)
	Low white cell count (1)

*The number in brackets is a count of the number of times each was mentioned in the treatment log.

**Table 2:** Specific symptoms affected by acupuncture (“*in vivo*” codes).

(i) Bloating (ii) Pitted oedema (iii) Headaches (iv) Eye pain (v) Hot flushes, night sweats (vi) Tiredness (vii) Anxiety (viii) Depression/low mood (ix) Feeling emotional (x) Kept me bubbly	(xi) Watery eyes (xii) Constipation (xiii) Sore throat (xiv) Nausea (xv) Tight chest (xvi) Reflux (xvii) Insomnia (xviii) Restless legs (xix) Achy joints (xx) It helped me relax
